# Hypodermic Administration of Medical Agents

**Published:** 1867-05

**Authors:** Wm. A. Greene

**Affiliations:** Americus, Ga.


ATLANTA

### Medical and Surgical Journal.

NEW SERIES.

Vol. VIII. MAY, 1867. No. 3.

### ORIGINAL COMMUNICATIONS.

### ARTICLE I.

*Hypodermic administration of Medicinal Agents.* By Wm. A. Greene, M. D., of Americus, Ga.

I have been using Hypodermic injections for several months, with the most satisfactory results. But having no data to control me in doses, and no experience as to the remedies best adapted to this mode of administration, my progress has been necessarily slow. But if I should never make further progress, I am fully remunerated for all my trouble. I now enter the chamber of *suffering*, *knowing* that I have in my possession an *unfailing* remedy for *pain*. "Relieve me of my pain, Doctor," is the cry of the sufferer. With a Hypodermic syringe, this agonizing cry can be promptly, and without injury, hushed.

I have employed Hypodermic injections in all forms of neuralgia,—both local and general,—in hysteria, wakefulness, delirium tremens, rheumatism, gout, threatened miscarriage, puerperal peritonitis, fever, painful affections of the nerves, caused by injury; and in all cases where *pain* calls for immediate relief: and when its employment for some potent reason is not contra-indicated. I have not time to give my experience and results in the treatment of all these affections by the Hypodermic method, and will, therefore, give practical facts.

The medicines I have used, with their doses, for a single injection, are as follows:—

Morphiæ Acetas, from 1-6 to ½ grain.
Atropæ Sulphas, from 1-60 to 1-30 grains.
Liquor Opii Comp. (Squibb's) from 5 to 60 drops.
Veratrum Veride (Norwood's) from ¾ to 2 drops.
Sulph. Quinine, from 3 to 8 grains.
Tr. Cannabis Indica, from 10 to 20 drops.

I make it a rule to begin with a minimum dose, establish a point of tolerance, and increase the number of drops as circumstances require. For general use, I prefer Dr. Squibb's comp. liquor of opium, which contains one grain of morphia to one hundred drops of the medicine; but when the *anodyne* effects are equal to officinal laudanum, the minimum dose is five drops; and can be extended to sixty drops at a single injection. I find it much less apt to produce nausea than any of the preparations of opium, and can be borne by the most delicate female.

To produce a quicker and more powerful effect, I employ the following solution of acetate of morphia:—

℞—Acetate Morphia, gr. xxiv.
Dist. Water, ℥i.
Acetic Acid, qs.—M.

Inject from 5 to 10 drops.

If I wish to produce a *still more* speedy and powerful effect, I combine with the dose of morphine a few drops of solution of atropia, as follows:—

℞—Sulphate Atropia, gr i.

Dist. Water, ʒiv.

Acetic Acid, qs.—M.

Inject from three to eight drops.

Atropia is a very powerful drug, and must be used very cautiously. While the morphine and atropia act well when combined, yet one is an antidote for the other. For instance: if you inject an over-dose of the atropia, you can counteract its effects in a moment with an injection of morphine; and *vice versa.* This is very singular but *true*; and should be remembered; for I have frequently given an over-dose of each, not knowing how much my patient would tolerate.

The tr. verat. veride must be used *very cautiously.* My friend Dr. G. F. Cooper, of this city, injected in the arm of a young man, suffering from ordinary fever—pulse 112—three-fourths of a drop of veratrum and four and one-half drops of the solution of morphine, which produced distressing nausea in ten minutes, and its full constitutional effects in twenty minutes; and no more was given or required during that paroxysm of fever.

In a case of profound coma, under my care, following a congestive chill, I injected two drops, which produced violent vomiting in five minutes, and full constitutional effects in fifteen minutes. The patient was a strong and robust youth of 18 years. He was completely relieved; and with large doses of quinine, recovered in a few days. I believe when tested, that veratrum will prove a most powerful auxiliary in the treatment of neuralgia, injected under the skin, at the *painful point.*

Sulphate of quinine acts promptly in doses from three to eight grains. I use the following solution:

℞—Sulph. Quinine, grs. xxx.
Sulph. Acid, gtt. x.
Water, ℥ ss.—M.

Tr. cannabis indica acts well in doses from ten to twenty drops. I have but little experience, as yet, with it.

From my experience, the Hypodermic administration of medicine commends itself above any other method, for the following reasons:—

1st—The amount received into the system is accurately known; every particle that is *injected* is *absorbed;* which is not the case in stomachic doses. For instance: if we introduce one-sixth of a grain of morphine beneath the skin, the effect that follows is that of the whole one-sixth; but if the same quantity is introduced by the stomach or rectum, the effect produced is only equal to the quantity *absorbed.*

2d—*Rapidity* of absorption is a great advantage of Hypodermic injections. For when introduced through the stomach, remedies have to pass through the *portal system* before reaching the general circulation.

3d—There are no circumstances under which it cannot be administered when indicated. Because the medical agents tastes badly, is nauseating or bitter, or the patient being delirious, refuses medicine altogether, or is unable to open his mouth or move the jaws, as in tetanus, *we can inject it under the skin.*

4th—We get a local and general eflect at the same time, which makes it particularly advantageous in neuralgia, where we have both a local and a general disorder.

5th—Persons who will not tolerate any of the preparations of opium by the stomach, will receive it kindly, and bear it charmingly when introduced sub-cutaneously. This *alone* should recommend it to the attention of every physician, as of incalculable value. And, again, the constipation and head symptoms, which usually follow the internal administration of the drug, are not to be apprehended. I will here mention two cases as demonstrating this point:—

Case 1.—Rev. A. A. Robinson, of this city, age about fifty, had his thigh fractured at middle of upper third, Dec. 10th, 1866. After Liston's long splint had been applied, and I had left him, thinking he would rest well from the chloroform he had taken, until I should see him again, he became restless, and was suffering so much, the nurse administered about one-quarter grain of morphine by the stomach, which produced excrutiating pain in the region of his stomach, violent nausea, and great nervous derangement, which he informed me was the invariable effect of any of the preparations of opium upon his system, when taken by the mouth. Soon as I reached his bed-side, I introduced under the skin of his arm ten minims of the solution of acetate of morphine, which brought complete relief in ten minutes; and he rested quietly for the next twelve hours. His peculiar nervous disposition, and circumstances surrounding him at the time of the accident, (being upon the eve of removing to another state) made him unusually restless and impatient, and, consequently, illy prepared for the quiet and composure required for a good recovery. Under these circumstances, it became necessary to administer the Hypodermic injections daily, sometimes morning and evening, for five weeks. During all this time there was no unusual constipation, no nausea, no loss of appetite, no unpleasant head symptoms, no *colic*; nor anything to retard a natural recovery from such an injury. Smith's Anterior Splint was used after the inflammatory symptoms subsided; and there was no *shortning*—a most fortunate and happy result, attributable to the quiet and rest produced by the Hypodermic injections. He could not bear opium or any of its preperations by the stomach; and his suffering must have been very great, coupled with a bad recovery, but for the Hypodermic injections; and so convinced was he of this fact, that he would not leave for his new home in Southern Florida, until a Hypodermic Syringe was ordered for him, and he instructed in its use.

Case 2.—Mrs. ———, age 25, of this City, was suffering from acute articular rheumatism, and when called to see her, remarked, so soon as I entered her room, "Doctor, you must not give me opium: it makes me crazy, and vomits me all next day." The disease was located in her wrist and fingers of right hand, and had resisted counter-irritants, blisters, and the usual general constitutional treatment for several days. The great pain and loss of sleep for several days, had produced much prostration. I at once injected under the skin of the affected wrist twenty minims of Squibb's liquor opii comp.; and in ten minutes she was relieved of all her pain; and in twenty minutes, was in a sound sleep, which lasted twelve hours. She awoke much refreshed; and the injections were contined at lengthened intervals,—at same time giving her colchicum and iron for ten days,—when she was dismissed, cured. The prompt action of occasional saline cathartics was not interfered with by the injections; neither did she *know* that she was taking *opium.*

6th—Finally, the remedy can always be at hand. The syringe is in a case, which contains a drachm vial, and can be carried in your vest pocket. No physician should be without one. The day will come when every physician will carry his Hypodermic syringe and morphine solution as religiously as our respected fathers once carried their lancet. What an amount of suffering could have been saved in our late war, if the Hypodermic administration of medicine had been generally known and used! Side by side with the discoverer of cloroform, and its adaptation to practical purposes, should be placed the man who first introduced to the notice of the profession this mode of the administration of remedial agents. In America, this credit is due Dr. Antoine Ruppaner, of Boston, whose indefatigable labors have enabled him to present to the profession a neat little work on Hypodermic injections; and from whose writings upon the subject I received my first impressions; and from whom I have drawn largely for the views contained in this communication. The afflicted of every section will "rise up and call him blessed."

The instrument I use is made by Tieman & Co., of N. Y., and consists of a graduated glass barrel, with a brass screw-piston, though so arranged that it can be worked as an ordinary P. P. syringe. A *two drachm* syringe is the most convenient size for ordinary purposes. Accompanyin the syringe, in same case, are two hollow needles, which ought to be very sharp. The finer the needle the better, for obvious reasons.

The operation is simple. Take hold of a fold of the skin, with the left index finger and thumb, so as to make the part beyond the fingers *tense*; then pass the needle through it with a quick movement. Throw in the solution slowly, and press the finger gently over the puncture for a moment after withdrawing the point of the syringe, so as to prevent the escape of any fluid, and to prevent bleeding. My friend, Dr. Geo. F. Cooper, of this City, suggests that the *point* be allowed to *remain* introduced under the skin, and the syringe detached, in cases where we are not well satisfied as to the dose required to produce the required effect, so that said dose may be *increased* without having to *re-puncture* the skin. This is a good suggestion, since it saves the patient the pain and fear of a second puncture; and we have to wait but a few moments to settle the question.

There is a diversity of opinion as to the *point* of injection—whether at the *painful point* or any other point. Some benefit will be experienced by introducing the medicine *any where* in the cellular tissue. I have not time to enlarge here; but my own experience is in favor of *locolization* of the injection, especially in neuralgia. I think it requires a smaller dose; and the effect is mnch more satisfactory and permanent.

In introducing the instrument, great care is required not to pierce a blood-vessel or wound a nerve. A correct knowledge of anatomy and caution, will prevent any accident of this kind. Neither should the same point be punctured *twice* in succession,—but immediately above it or below it. Frequent punctures at the same point endangers abscess. Vomiting results from an over-dose of the preparations of opium or atropine. This you will control easily with sul. nit. bismuth, or oxalate of cerium. I prefer the latter.

I will bring this hastily written article to a close, by stating a few cases from my note-book—treated with Hypodermic injections. For my own justification, I feel it my duty to state that this communication has been written under pressing professional engagements, and with an honest desire that the attention of the profession may be directed to this new and unexplored field of medicine, that promises such happy and beneficial results..

Case 1.—Mrs. P.——of Americus, Ga., aged forty, mother of several children, consulted me first of December, 1866, for severe neuralgia of the face and head, of twenty years standing. Pain confined to the left side, extends to the sagital suture, of a dull heavy nature, almost constant, but at one time more severe than at another; has lost the sight of the left eye from amaurosis several years since. She also complains of a circumscribed pain around the ear. The lachrymal branch of the ophthalmic division and the potio dura is evidently involved.

She had taken and "worn-out," quinine, morphine, strychnine, valerian, iron, and in fact, the catalogue of neuralgia remedies. I advised the Hypodermic injection. She did not consent to it until December 25th, when I was sent for. I found her in one of her most painful paroxysms: I injected fifteen drops of the solution comp. liq. opii at the palpebral point; and in ten minutes Mrs. P——was at ease. Five days afterwards the operation was repeated; again four days later; and occasionally afterwards at longer or shorter intervals, as her pain required ,for six weeks. The patient taking at same time, the hypophosphite of soda, in drachm doses, three times per day, and citrate of iron and strychnine in proper doses.

No return of neuralgia at present date of writing—March 28, 1867—being her longest period of relief since her attack. Her general health much better, and fast improving.

Although sufficient length of time has not elapsed in this case, to test the permanency of relief, yet enough has been achieved to warrant the assertion, that if she is not *cured*, her disease is at least so much mitigated, as to make her life tolerably comfortable, with a strong hope and good prospect of permanent relief. I shall watch this case with much interest.

Case 2.—Mrs.——of this city, aged twenty; nervous temperament, and very excitable; six months gone in pregnancy with first child,—sent for me December 18th, 1866, in great haste, the messenger stating she was threatened with miscarriage. Arrived in her room ten minutes before eleven o'clock, P. M. Found her suffering with severe bearing-down pains at intervals of every five minutes.

At eleven o'clock, introduced ten minims of solution acetate morphine, at the insertion of the deltoid muscle of right arm. In fifteen minutes, said she felt *very comfortable*, and in fifteen minutes more was sound asleep. She awoke at two o'clock, complaining of slight pain, when I introduced five minims more of the solution, and left her. I saw her no more until 11th March, when I was summoned to attend her in labor. After a painful and tedious labor of thirty hours, she gave birth to a large child.

Three days after her delivery, she was violently attacked with puerperal peritonitis. All the *pain* of this terrible disease, in her case, was completely controlled by the Hypodermic administration of comp. liq. opii, whenever required, which in no wise interfered with the proper treatment. She is now out of danger, (March 23d) making a rapid recovery, and suffered less than any patient I ever treated, without the Hypodermic injections.

This lady could not take, in any quantity, any of the preparations of opium, without the most distressing vomiting and wakefulness. My friend, Dr. Cooper, visited this case with me, and witnessed the charming effects of the remedy.

I think this a most interesting and instructive case.

Case 2.—Mr.——, aged thirty, of small stature, nervous temperament, consumptive, addicted to drinking, had suffered from an attack of delerium tremens for two days, when I saw him. He had suffered frequently from the disease.

Morphine, valerian, chloroform, and veratrum had failed to control his delerium. He would not go to bed; attempted to move away from his friends; refused to allow me to examine him in any way; swore the house was on fire, and the devil was trying to throw him into it, etc., etc. I found persuasion useless. I had him confined, and introduced at the first point I could (which was the top of his shoulder) a mixture of ten minims of the solution of acetate of morphine and five minims of the solution of sulphate of atropia. In fifteen minutes, he complained of feeling tired; was put to bed, and went to sleep; once or twice made feeble attempts to get up, but was easily controlled.

In three hours, repeated the operation without the atropine.

Heard no more from him for three or four days, when I saw him on the street.

There is no physician who does not dread the annoyance of a case of delirium tremens. Well, you need have no *further* dread, if you will provide yourself with a Hypodermic syringe. I have treated several cases with the above gratifying result.

Case. 4. Feb. 13th, 1867. I was called to visit Mr. G., two miles from town; he is 22 years old, well built, and regular habits; found him suffering excrutiatingly from bilious colic of three hours duration; he had been freely vomited with mustard; had taken laudanum, morphine, chloroform, whisky, &c., &c., to no purpose. His friends thought he must certainly die. I at once introduced twenty minims of comp. liq. opii and five minims of solution of atropine in the arm. In five minutes, considerably relieved; and in ten minutes, *completely at ease.* Directed three comp. cathartic pills at bed time, and left him. He was attending to his business next day.

Case 5. Was called to see Mr. S., of this City, January 15th, 1867; he is 38 years old, strong and robust; found him suffering from an attack of remittent fever; most intense pain in head, back, and limbs; very irritable stomach, and great restlessness; pulse 130, and full; thirst very great, but vomits every time he attempts to take water; has complete disgust for medicine, and is clamorous for relief. I confidently promised to put him at ease in a few moments. I injected twenty minims comp. liq. opii under the skin of his arm; and in twenty minutes pain was charmed away, and sleep came when least expected. The stomach received and appropriated the remedies; strength was not made subordinate to debilitating measures; and convalesence was more rapid than ordinarily.

Case 6. Mr. R., of this City, had suffered for several months from *bone-felons* and carbuncles. He consulted me Dec. 18, 1866,—suffering from a palmer abscess of the right hand, and a carbuncle on back of his neck. He was in a distressed condition; was emaciated and anæmic from frequent attacks of chills and fever; had not been able to sleep for several nights; said he had been "cut so much" he could not again submit to it; and was afraid of chloroform, because he was consumptive. I advised him to submit to the required operation under the influence of the Hypodermic injection of morphine, which I thought would at least make the pain *bearable.* He agreed to it; and I injected ten minims of the solution of morphine and five minims of the solution of atropine under the skin of the back of the affected hand. In five minutes he was so much effected as to be put to bed. I immediately made a deep incision through the palmer facia, turning out a considerable quantity of puss; also at same moment opened the carbuncle on back of neck. He manifested but little pain or concern for the operation. Sleep was irresistible, which lasted several hours. He awoke considerably nauseated, which was speedily silenced with half grain of acetate of cerium.

I have had no other opportunity of testing the efficacy of Hypodermic injections in relieving the pain of minor operations. In this case it certainly acted well, and if I had waited until my patient was sound asleep, I doubt if he had felt the pain at all,—as the pressing, syringing, and dressing, after he was asleep, did not disturb him.

It will be found invaluable in relieving pain and nervous irritability in all surgical accidents. It has also been found to prolong the anæsthesia from chloroform. I now invariably inject from one-quarter to one-half grain of morphine, when I expect my patient to be continued under chloroform for a length of time. The attention of the profession is respectfully called to this fact; and I hope those having larger opportunities than myself will test its efficacy, and report their experience to the profession.

This article has been unintentionally spun out to an uninteresting length. I only hope to call the attention of my professional brethren to this interesting and important subject; and if they will only try it, I have no misgivings about their experience coinciding with mine. Its field of application is not limited now, as formerly, to the various forms of neuralgia; but its usefulness has been demonstrated in various other diseases,—thus opening up a most extensive field for investigation.

